# Use of Antigen Combinations to Address Complex *Leishmania*-Seropositivity Patterns in Dogs Living in Canine Leishmaniosis Endemic Regions of Portugal

**DOI:** 10.3390/microorganisms10102018

**Published:** 2022-10-12

**Authors:** Carla Silva Lima, Sofia Esteves, Inês Costa, Hugo Brancal, Clara Lima, Célia Amorim, Luís Cardoso, Nuno Santarém, Anabela Cordeiro-da-Silva

**Affiliations:** 1 Instituto de Investigação e Inovação em Saúde, Universidade do Porto, 4200-135 Porto, Portugal; 2 Instituto de Biologia Molecular e Celular, Universidade do Porto, 4200-135 Porto, Portugal; 3 Serviço de Microbiologia, Departamento de Ciências Biológicas, Faculdade de Farmácia da Universidade do Porto, 4050-313 Porto, Portugal; 4 Clínica Veterinária da Covilhã, 6200-293 Covilhã, Portugal; 5 Faculdade de Ciências da Saúde, Universidade da Beira Interior, 6200-506 Covilhã, Portugal; 6 Departamento de Ciências Veterinárias, Escola de Ciências Agrárias e Veterinárias, Universidade de Trás-os-Montes e Alto Douro (UTAD), 5000-801 Vila Real, Portugal; 7 Centro de Ciência Animal e Veterinária (CECAV), UTAD, 5001-801 Vila Real, Portugal; 8 Laboratório Associado para Ciência Animal e Veterinária (AL4AnimalS), Universidade de Trás-os-Montes e Alto Douro (UTAD), 5000-801 Vila Real, Portugal

**Keywords:** Canine leishmaniosis, clinical infection, DAT, ELISA, *Leishmania* antigens, rK39, serological tests, subclinical infection

## Abstract

Canine leishmaniosis (CanL) is a vector-borne disease caused by *Leishmania infantum*. Infection in dogs can result in a disease with non-specific clinical signs or in a subclinical condition. Infection diagnosis is crucial to guide public health measures considering the zoonotic potential of *L. infantum*. Serological approaches to detect infection with a reduced antigen panel potentially limit the quality of the information obtained. To evaluate the impact of using distinct antigens in a serological survey, a cohort with 390 dogs from endemic regions in Portugal was subjected to a serological evaluation using ELISA and DAT. Using ELISA, six *Leishmania*-specific antigens in conjunction with a non-related antigen, *Escherichia coli* soluble antigens, were evaluated. The global seroprevalence was 10.5% for DAT and 15.4 to 23.1% for ELISA, depending on the antigen for the latter. Still, only 8.2% of the animals were seropositive to all *Leishmania*-specific antigens. Importantly, a further 31.0% presented antigen-dependent seropositivity. Considering this observation, a serological score system was proposed and validated to address the complex serology results. With this system, the overall dog seropositivity was 26.9%. This work highlights the limitations of single-antigen serological surveys and presents an approach that might contribute to the establishment of CanL-specific serological profiles.

## 1. Introduction

Canine leishmaniosis (CanL), caused by the protozoan parasite *Leishmania infantum*, is a vector-borne parasitic disease of dogs (*Canis lupus familiaris*). Female sand flies are the vectors responsible for parasite transmission. In geographical areas where susceptible sand flies are present, dogs are the main reservoir of *L. infantum* and represent an increased risk for zoonotic transmission to humans [1,2,3,4]. *L. infantum* infection in dogs can be associated with non-specific clinical signs (alopecia, conjunctivitis, cutaneous lesions, skin ulceration, lymphadenopathy, cachexia, onychogriphosis, hyperkeratosis, and anorexia), or can assume a subclinical form [1,2,5,6]. While clinical infections in dogs present high antibody titres, subclinical infections can present low, fluctuating, or even absent *Leishmania*-specific antibody titres [7]. Although the impact of the transmission associated with subclinical infections is a matter of debate, dogs with these subclinical infections can still act as reservoirs that are competent to transmit the parasite to the sand flies [8,9]. Therefore, their detection has a significant role in the management of leishmaniosis. The detection of parasite DNA by PCR, although highly specific, presents variable sensitivity depending on the time post-infection, infection progression, and the type of sample analysed [10]. The evaluation of *Leishmania*-specific antibody responses by serological evaluation is highly used, not only for CanL diagnosis, but also in epidemiological surveys. Enzyme-linked immunosorbent assay (ELISA) and the direct agglutination test (DAT), presenting high sensitivity and variable specificity, are methods suitable for mass-screen epidemiological studies [11,12]. The technical disadvantages of DAT are the necessity of serial dilutions of the samples to determine the endpoint titre and a long incubation period of 18 h. An inter-observer discrepancy in DAT readings is also possible, requiring trained technicians [13,14,15]. ELISA tests are ideal for serodiagnosis, being rapid, cheap, user-friendly, and allowing for the screening of large numbers of samples [16,17]. The main limitation of ELISA, when using total parasite extract, is the cross-reactivity reactions with other pathogens that often co-exist in endemic areas leading to inconsistent results [18,19]. The use of recombinant proteins or their combination in ELISA assays is a strategy that can improve the global sensitivity and specificity of the test, preventing cross-reactivity [11,20,21,22]. The reliability of serological techniques in CanL suspect animals is limited with 50% of the animals presenting antigen-dependent seroreactivity [23]. Therefore, the evaluation of antigen-dependent seroreactivity in the context of epidemiological screenings is a necessity. These screenings are often performed using reduced antigen sets and might generate inaccurate data due to cross-reactivity or inadequate cut-offs. The level of antigen-dependent serological reporting is a concern and should be clarified for better integration of serological data in epidemiological settings. To address this question, we evaluated the potential of six different *Leishmania*-specific antigens: soluble promastigote *Leishmania* antigen (SPLA), recombinant antigens (LicTXNPx, rK39, rK28, and rKDDR), a mixture of recombinant antigens (LAM) [20] in conjunction with a non-related antigen, *Escherichia coli* soluble antigens (SECA) [24]. Crude promastigote antigens, the SPLA, have been described as highly sensitive for subclinical and clinical infections, but with a lower specificity [25,26]. The rK39, rK28, and rKDDR are all kinesin-derived recombinant proteins with proven value for the serodiagnosis of CanL [27,28]. LicTXNPx is a recombinant protein that demonstrated an increased capacity to detect subclinical infections [20,29]. LAM is a mixture of rK39 and LicTXNPx optimized to detect subclinical infections without compromising the sensitivity for clinical CanL [20]. Thus for the first time, serological data with established antigens, kinesin-related antigens rK39, rK28, and rKDDR will be compared to SPLA and other antigens of interest, like LicTXNPx, to address the seropositivity profile of 390 dogs from a CanL-endemic area in Portugal. This will permit the evaluation of possible restraints of single antigen serological surveys and highlight the importance of *Leishmania*-specific serological signatures as an alternative to address complex serological data. Moreover, the data provided will contribute to further validation of the serological performance of several *Leishmania*-specific antigens, using the same ELISA technical approach.

## 2. Materials and Methods

### 2.1. Samples

Group Leish+ (n = 29): sera from dogs living in geographical regions of Portugal where CanL is endemic, collected in a veterinary centre, or during anti-rabies campaigns, and which presented at least two clinical signs (CS) compatible with the disease (lymphadenomegaly lymphadenopathy, alopecia, dermatitis, skin ulceration, keratoconjunctivitis, onychogryphosis, lameness, epistaxis, anorexia, and weight loss). These animals were also seropositive for anti-*Leishmania* antibodies by the direct agglutination test (DAT) (cut-off titre = 400) and/or positive for the presence of amastigotes in bone marrow or lymph node aspirates.

Group Leish- (n = 121): sera from dogs that visited a veterinary centre in a Portuguese region that is considered to be non-endemic for CanL. All dogs were seronegative by DAT (titer < 100).

Group Portugal (PT) (n = 390): sera from dogs with unknown serological status, mostly collected in dog kennels and a veterinary centre in five endemic areas in the north and centre of Portugal (Lamego, Covilhã, Fundão, Proença-a-Nova, and Idanha-a-Nova) with the legal responsibility and owners’ informed consent, respectively, during June 2011. The PT group contains six groups (A, B1, B2, C, D, and E) defined according to three parameters: clinical data-presence of CS, seropositivity by DAT, and molecular detection of *Leishmania* DNA by PCR (Table 1). Animals from subgroup A presented at least one CS compatible with leishmaniosis (alopecia, conjunctivitis, cutaneous lesions, skin ulceration, lymphadenopathy, cachexia, onychogryphosis, hyperkeratosis, and anorexia) and DAT positive test (PCR could be positive or negative). Subgroup B1 and subgroup B2 dogs presented no CS and were DAT positive. Subgroup B1 presented positive PCR and subgroup B2 was negative for PCR. The B1 and B2 subgroup are part of the B group, animals with a serological response, DAT positive, but without clinical signs of disease. Subgroup C included the animals that presented no CS, negative DAT, and positive PCR. Subgroup D did not present CS and was negative for DAT and PCR. Finally, animals in subgroup E presented CS but had negative DAT and PCR.

### 2.2. Direct Agglutination Test

The DAT protocol was performed as described by Schallig et al. [30]. Previously, serum samples were diluted in saline (0.9% NaCl) containing 1.56% of β-mercaptoethanol. Serial dilutions were made in V-shaped microtitre plates (Greiner Bio-One GmBh, Frickenhausen, Germany) starting with 1:100 until the maximum of 1:102 400 for each sample, followed by an incubation of 1h at 37 °C. Next, 5 × 10^7^ freeze-dried *L. donovani* promastigotes (MHOM/SD/68/1S)/mL were suspended in 5 mL of physiological saline (0.9% NaCl), according to the manufacturer’s instructions (Amsterdam University Medical Centres, Academic Medical Centre at the University of Amsterdam, Department of Medical Microbiology, Section Experimental Parasitology, Amsterdam, The Netherlands). After dispensing 50 µL of DAT antigen per well, plates were incubated for 18 h at room temperature. Results obtained with DAT are expressed as an antibody titre, i.e., the reciprocal of the highest dilution at which agglutination (large diffuse blue mats) is still clearly visible after 18 h incubation at room temperature. A cut-off titre of 400 has been chosen to maximize the sensitivity and specificity of the test. Positive controls for the DAT consisted of serum samples from dogs diagnosed with leishmaniosis. DAT titres in these samples ranged between 51 200 and ≥102 400. Sera from dogs living in areas where leishmaniosis is not endemic were used as negative controls.

### 2.3. DNA Isolation

DNA was extracted from blood samples using the E.Z.N.A Blood Mini Kit (Omega Bio-teK, Norcross, GA, USA), following the manufacturer’s instructions. The concentration and quality of DNA obtained from tissues was determined with a NanoDrop (Thermo Fisher Scientific, Wilmington, DE, USA), and then stored at −20 °C until use.

### 2.4. Polymerase Chain Reactions

Using small subunit ribosomal ribonucleic acid (SSUrRNA) as the target gene, the first amplification was performed using the primers R1 (5′GGTTCCTTTCCTGATTTACG 3′) and R2 (5′GGCCGGTAAAGGCCGAATAG3′), specific for Kinetoplastida. The Nested-PCR protocol was adapted from Cruz et al., 2002 [31]. Briefly, the volume of the reaction was 50 µL, containing 10 µL of genomic DNA, 29.7 µL of nuclease-free water (Invitrogen, Carlsbad, CA, USA), 15 pmol of R1 and R2 primers, 10 mM deoxynucleoside triphosphates 10 mM MgCl_2_, and 1.4 U of NZYTaq DNA polymerase, (NZYTech, Lisbon, Portugal). The PCR program included initial denaturation at 94 °C for 5 min, followed by 30 cycles of 30 s at 94 °C, 30 s at 60 °C, and 30 s at 72 °C, with a final extension at 72 °C for 5 min. Samples positive for SSUrRNA revealed a 603 bp product.

The second reaction was performed using R3 (5′TCCCATCGCAACCTCGGTT3′) and R4 (5′AAAGCGGGCGCGGTGCTG 3′) *Leishmania*-specific primers in order to re-amplify the PCR product of the first reaction. These PCR products were diluted (1/40) with nuclease-free water. The volume of the reaction was 25 µL, containing 10 µL of the first PCR product (diluted 1/40), 10.1 µL of nuclease-free water (Invitrogen, USA), 15 pmol of R1 and R2 primers, 10 mM deoxynucleoside triphosphates 10 mM MgCl_2_, and 1.4 U of Taq polymerase (NZYTaq DNA polymerase, nzytech, Portugal). The PCR program included initial denaturation at 94 °C for 5 min, followed by 30 cycles of 30 s at 94 °C, 30 s at 65 °C, and 30 s at 72 °C, with a final extension at 72 °C for 5 min. Samples positive for *Leishmania* revealed a 353 bp product. Amplification products were visualized after electrophoresis in 2% agarose gel with a 1000 bp DNA ladder (NZYDNA Ladder V, NZYTech, Portugal) and stained with 0.1% of ethidium bromide. 

### 2.5. Antigens 

For SPLA production, *L. infantum* MHOM/MA/67/ITMAP-263 promastigotes were cultivated as previously described [32]. Parasites with 5 days of culture were washed three times with phosphate-buffered saline (PBS), pH 7.4, and centrifuged at 3500× *g*, 10 min, at 4 °C. The pellet was suspended in PBS containing 1 mM phenylmethylsulfonyl fluoride protease inhibitor and submitted to 10 freeze-thaw cycles for rupture of the parasites. This suspension was centrifuged at 13,000× *g*, 30 min, at 4 °C and the supernatant was recovered, quantified by DC (detergent compatible) protein assay (Bio-Rad Laboratories, Hercules, CA, USA), and stored at −80 °C in single aliquots. The recombinant protein LicTXNPx was purified by affinity chromatography on a Ni-NTA column (Qiagen, Hilden, Germany), as described in previous reports [33], and is obtained as a recombinant protein containing six histidine residues at its N-terminal. LicTXNPx was quantified by DC (detergent compatible) protein assay (BioRad), and stored at −80 °C in single aliquots. The rK39 and rK28 lyophilized antigens, obtained from Dr. Steven Reed (Infectious Disease Research Institute, Seattle, WA, USA), were suspended in deionized and 0.22 μm membrane-filtered H_2_O, quantified and stored at −80 °C in single-use aliquots. The recombinant protein kDDR, provided by Dr. Ricardo Fujiwara (Universidade Federal de Minas Gerais, Belo Horizonte, Brasil), was quantified by DC (detergent compatible) protein assay (BioRad), and stored at −80 °C in single aliquots. *Leishmania* antigen mixture (LAM) was prepared as described by Santarém *et al.* [20], combining LicTXNPX (1µg/mL) and rK39 (4 µg/mL) and storing the pre-prepared mixtures in single dose use at −80 °C. 

### 2.6. Enzyme-Linked Immunosorbent Assay (ELISA)

Ninety-six-well flat-bottom microtiter plates (Greiner Bio-One, Germany) were coated with the antigen in 50 µL of 0.05 M carbonate buffer, pH = 9.6. The antigen concentrations used were 10 µg/mL of SPLA, 3 µg/mL of LicTXNPx, 1 µg/mL of rK39, 4 µg/mL of rK28, 3 µg/mL of rKDDR, and 5 µg/mL of LAM (1 µg/mL of LicTXNPx + 4 µg/mL of rK39). Plates were incubated O/N at 4 °C and blocked with 200 µL of PBS-low-fat-milk (3%) at 37 °C for 1 h. Next, plates were washed with PBS-Tween 0.05% (PBS-T), and the sera, positive, and negative controls, diluted at 1:1500 in PBS-T were dispensed in triplicate (100 µL/well) and incubated at 37 °C for 30 min. After a washing step, 100 µL/well of conjugate—secondary anti-dog IgG antibody conjugated with horseradish peroxidase (Sigma, USA)—diluted at 1:1176.5, was added and the plates were incubated at 37 °C for 30 min. Plates were washed and incubated with 0.5 mg/mL of o-phenylenediamine dihydrochloride (Sigma-Aldrich, St. Louis, MO, USA) for 10 min in dark. The reaction was stopped with 50 µL/well of HCl (3 M). Absorbance was read at 492 nm in an Synergy 2 automatic reader (Agilent, Winooski, VT, USA). All samples and antigens were assayed in at least two independent assays.

### 2.7. Score System

The proposed score system was used as described by Lima et al. [24] with minor modifications. Optical densities of all samples were screened for seroreactivity using a threshold of 0.014. If a sample presented a seroreactivity of less than 0.014 was excluded from the cut-off approach and the score for that ratio was considered zero. This 0.014 is considered the technical limit for seroreactivity in our laboratory settings and was calculated using the average of the blanks and two standard deviations. Values less than 0.014 are not consistently seroreactive in technical replicates and, therefore, cannot be used in the analysis because they originate random ratios that are not dependent on seroreactivity. This was a modification from the proposed model [24] and was done to prevent artefact values for the ratios. Each sample OD at 492 nm was normalized by division with the corresponding cut-off value. The cut-off normalized values (n) were used to assess the ratio between the antigens: SPLA/SECA (ratio between nSPLA and nSECA), rK39/SECA (ratio between nrK39 and nSECA), and rK39/SPLA (ratio between nrK39 and nSPLA). A score evaluation method was established based on the cut-off values inferred from the ROC curves for the ratios and the single antigens. A binary score (1 or 0) was applied to represent seropositivity (arbitrary value of 1) or seronegativity (arbitrary value of 0) to the five serological parameters previously established and whose cut-off values were determined by ROC curve analysis: rK39, SPLA, rK39/SECA, SPLA/SECA, and rK39/SPLA.

### 2.8. Statistical Analysis

Receiver operating characteristic (ROC) curves were generated using sera from groups Leish+ and Leish-. A 95% confidence interval (95% CI) for the area under the curve (AUC) was considered. The quality associated with the AUC values was excellent for AUC between 0.9 and 1, very good for 0.8 to 0.9, good for 0.7 to 0.8, satisfactory for 0.6 to 0.7, and unsatisfactory for 0.5 to 0.6. Cut-off values were inferred through these curves for each antigen (by choosing the best compromise between sensitivity and specificity associated with the ROC curve), and values of sensitivity (Se) and specificity (Sp) were calculated for the samples for each group [34]. Optical densities of each sample were normalized by division with the corresponding cut-off value and the logarithm of this ratio was applied for graphical representation. For comparison of the OD between the subgroups, an ordinary one-way ANOVA with Tukey’s multiple comparison test was used, with single pooled variance. All statistical analysis was performed using Prism 9 for Windows version 9.4.0. The UPSETPLOT was done using the online ImageGP software [35]. Cohen’s kappa used for agreement quantification was calculated using the Giacomo Scarpellini–IdoStatistics software [36].

## 3. Results

### 3.1. Cut-Off Determination by ROC Curves

For all the antigens, SPLA, rK39, rK28, LicTXNPx, rKDDR, LAM, and SECA, a ROC curves analysis was performed using the Leish+ and Leish- cohorts (Table 2 and Supplementary Figure S1). All AUC for the *Leishmania*-specific antigens were associated with excellent quality (AUC between 0.9 and 1.0). The cut-off values used enabled the best compromise between sensitivity and specificity. For the non-specific antigen SECA, the calculated AUC was 0.79, a good quality model (Supplementary Figure S2). The antigen capacity to discriminate the Leish+ and Leish- cohorts were evaluated by the sensitivity (Se) and specificity (Sp) (Table 2). SPLA, rK39, rK28, and rKDDR presented 100 % Se and Sp. For LAM, although the Se was 99.2 %, the Sp was 100 %. LicTXNPx presented the lowest values of Se and Sp: 94.2 % and 93.1 %, respectively.

### 3.2. Serological Survey of PT Cohort

The 390 dogs from the PT cohort were clinically evaluated and tested by DAT and PCR. In the 390 dogs, the overall frequency of at least one CS compatible with CanL was 17.9 % (70/390) presented, 10.5 % (41/390) of the dogs were seropositive by DAT, and 6.1 % (24/390) were PCR-positive. On the other hand, 279 dogs did not present any of the above-mentioned traits. This information was used to divide the cohorts according to what is presented in Table 1. 

**Figure 1:**
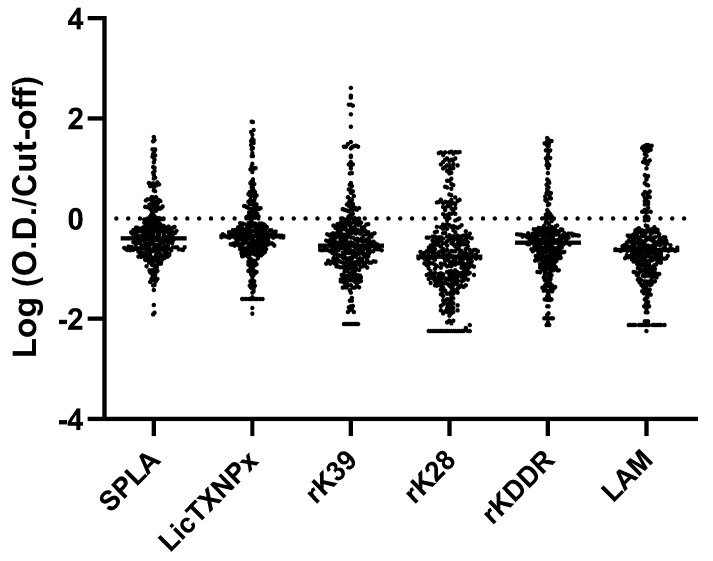
Representation of seroreactivity in PT group (n = 390) for the different antigens tested: SPLA, LicTXNPx, rK39, rK28, rKDDR, and LAM. Results are expressed as the logarithm of the optical density (OD) at 492 nm normalized by the cut-off value for each antigen. Every single dot depicts the average of two independent assays done in triplicate for an individual dog. The horizontal bar associated with each antigen data set is the median value. The dashed line represents the cut-off for all the antigens. Dots with positive and negative values represent seropositive and seronegative dogs, respectively.

The seroreactivity of the PT cohort to the described panel of seven different antigens tested by ELISA was evaluated using the average OD at 492 nm (Supplementary Figures S3–S5). The generic profile of seroreactivity associated with the *Leishmania*-specific antigens was similar with higher OD values for groups A and B1, intermediate for B2, and lower for C, D, and E (Supplementary Figures S4 and S5). This pattern was not evident for SECA, the overall seroreactivity was not significantly different in any of the subgroups (Supplementary Figure S3). All the six *Leishmania*-specific antigens presented significantly higher seroreactivity in the A subgroup compared to D, *p* < 0.0001 for SPLA, rK39, and LicTXNPx and *p* = 0.0414 for rK28, *p* = 0.0004 for rkDDr, and *p* = 0.0225 for LAM. In the PT cohort, the 492 nm OD values were normalized with the respective cut-off value for each antigen, inferred previously by the ROC curves in the Leish+ and Leish- cohorts, and logarithmized for better graphical interpretation (Figure 1).

The average seropositivity for the PT cohort was 18.15% (Table 3). The highest values of seropositivity were associated with the LicTXNPx antigen 22.3 % (87/390) and SPLA 21.5% (84/390). For rK39, seropositivity was 16.2% (63/390), for rK28 17.7% (63/390), and for rKDDR 15.6% (61/390). LAM presented a seropositivity percentage of 15.6 % (61/390).

To evaluate the distribution of seropositivity, the PT cohort was divided into six subgroups (A, B1, B2, C, D, and E), as previously described (Table 3). All dogs from subgroup A were seropositive for the six *Leishmania* antigens tested (13/13). The dogs from subgroup B1 were also seropositive for all *Leishmania* antigens tested, except for two that were seronegative only for LicTXNPx. In subgroup B2, the antigen associated with the highest seropositivity was rK28 with 82.6% (19/23) of seropositive dogs. For subgroup C, LicTXNPx with 38.5% (5/13) of seropositive dogs was the antigen associated with the highest seropositivity. In subgroup D, LicTXNPx with 15.4% (43/279) of seropositive dogs presented the highest seropositivity. The other 5 antigens presented lower: SPLA 10.8% (30/279), 5.4% for rK39 (15/279), 6.4% for rK28 (18/279), 4.3% for rKDDR (12/279), and 5.4% (15/279) for LAM. In subgroup E, SPLA with 26.3% (15/57) presented the highest seropositivity. When comparing E and D subgroups, except LicTXNPX 14.0% (8/57), all other antigens presented higher seropositivity when compared with group D, LAM 15.8% (9/57), rK39 and rKDDR 19.3% (11/57), and rK28 21.0% (12/57) (Table 3). Subgroup E was also associated with higher median seroreactivity when compared to D (Supplementary Figure S5). A higher median seroreactivity was also observed when comparing the E and D subgroups to the Leish- group used for ROC curve determination.

**Figure 2:**
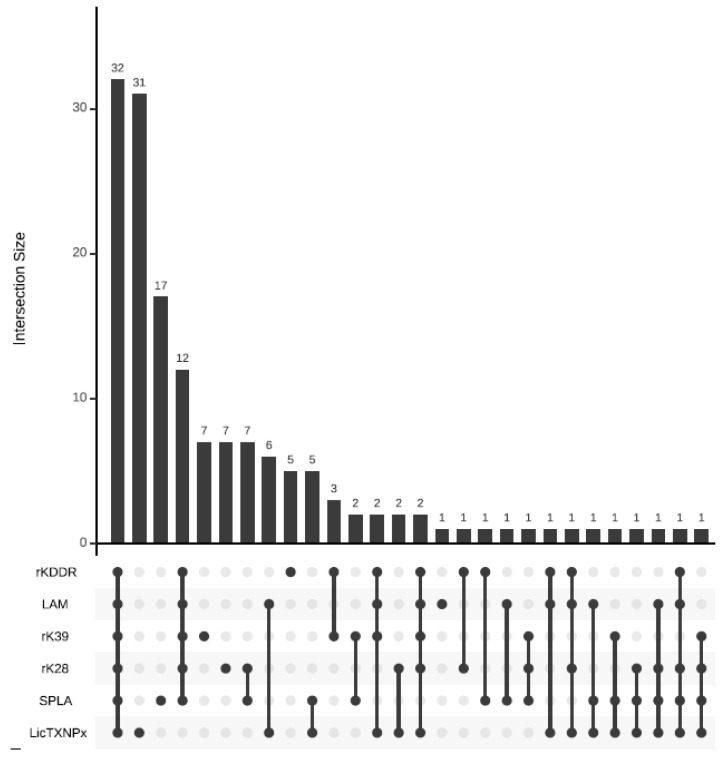
UpSet Plot depicting seropositivity intersect to rKDDR, LAM, rK39, rK28, SPLA, and LicTXNPx in the PT cohort. The black bars in the graph represent the absolute number of positive events of each intersection, associated with the six parameters evaluated. Under each black bar, the connecting line represents the intersection of the tested parameters. If no intersecting line is present it means that dogs only positive to one event are being quantified in the upper black bar.

**Figure 3:**
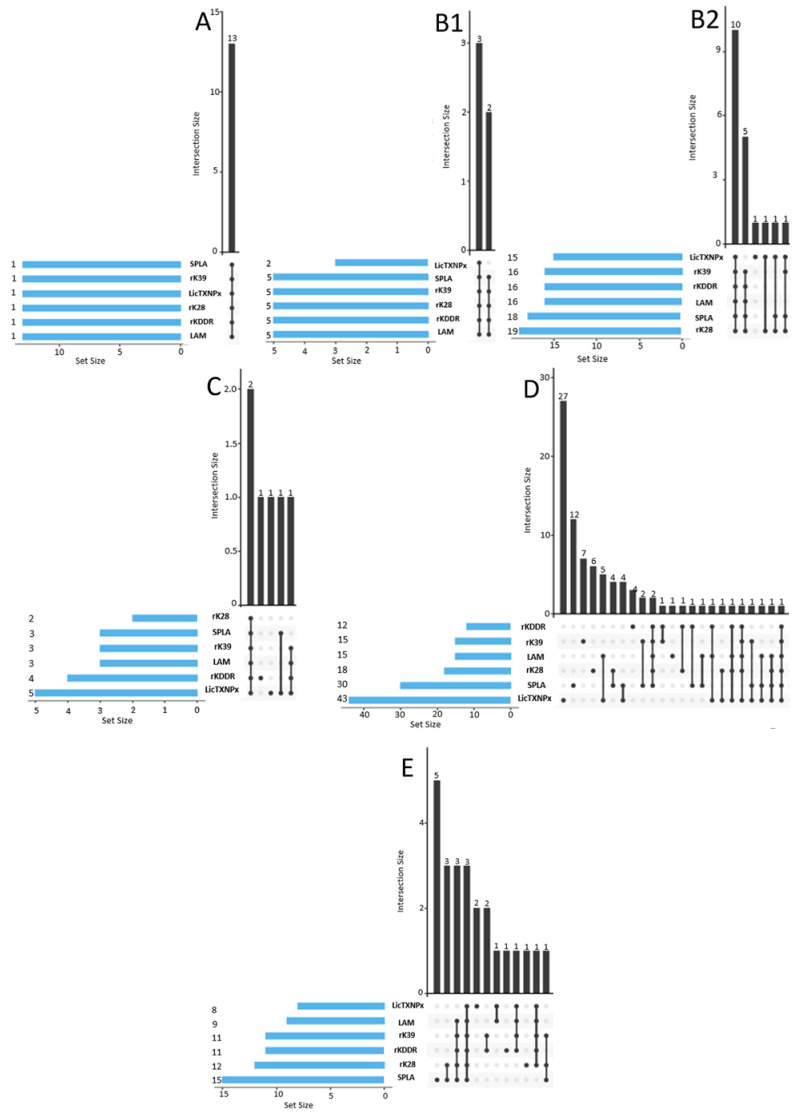
UpSet Plot depicting seropositivity intersect to rKDDR, LAM, rK39, rK28, SPLA, and LicTXNPx in the cohorts used to subdivide the PT cohort (**A**,**B1**,**B2**,**C**–**E**). The black bars in the graph represent the absolute number of positive events of each intersection, associated with the six parameters evaluated. Under each black bar, the connecting line represents the intersection of the tested parameters. If no intersecting line is present it means that dogs only positive to one event are being quantified in the upper black bar. To the left, the blue bars represent the absolute number of dogs that are positive for each individual characteristic evaluated. In each individual graphic the antigens are ordered by increasing number of seropositive animals.

Considering that the PT cohort’s average seropositivity was 18.5%, a more detailed evaluation of individual seropositivity was performed (Figure 2 and Figure 3 and Supplementary Table S1). It is worth noting that 31.0% (121/390) of the dogs presented a mixed serology profile. 

The most common seropositivity scenarios were: all antigens with 32 dogs, LicTXNPx only with 31, SPLA only with 17, and all antigens except LicTXNPx with 12 (Figure 2). The most common seropositivity combination in the PT cohort included the six antigens and was most common in A, B, and C subgroups (Figure 3).

Only four animals, one in subgroup D and three in E were seropositive to all antigens. LicTXNPx monopositivity was the most frequent scenario in subgroup D with 27 animals. SPLA monopositivity was the most frequent scenario in the E cohort with five dogs and was also present in dogs from the D cohort. The seropositivity to five antigens while being negative to LicTXNPx was mostly present in subgroup B with seven animals present, two in B1 and five in B2.

Considering the mixed positivity in the subgroups, dogs from subgroup A were positive for all antigens (Supplementary Table S1 and Figure 3). Regarding subgroup B1, 60% of the dogs (3/5) were positive for five out of six antigens, with 43.4% (10/23) of dogs from B2 presenting mixed serology. Considering subgroup C, 30.7% (4/13) presented mixed serology. For dogs without evidence of infection, subgroups D and E, the percentage of mixed serology was 30.9% (104/336). This percentage is higher compared to Leish-, with just 5.8% (7/121) of mixed serology. To quantify the degree of accordance of the seropositivity to the antigens seropositivity Cohen’s kappa coefficients (k) were calculated (Supplementary Table S2). The antigens with the highest k value were rKDDR and LAM, with a substantial agreement between them (k = 0.8058). All the other *Leishmania*-specific antigens analysed presented a fair to moderate agreement (Supplementary Table S2). This diminished agreement between tests is even more evident when the analysis is performed only with dogs without evidence of infection, subgroups D and E (Supplementary Table S3). The k-coefficient values decreased for all comparisons made. As expected the SECA antigen presented no agreement with the other antigens.

### 3.3. Score Systems Using Ratio Approaches

As an alternative to simple seropositivity evaluation, a scoring system, previously described by Lima et al. [37], using the serological response to SPLA, rK39, and SPLA to calculate antigen ratios, SPLA/SECA, rK39/SECA, and SPLA/rK39, with predictive capacity in the context of CanL was used (Supplementary Table S4 and Supplementary Figure S6). The ratios SPLA/SECA, rK39/SECA, and SPLA/rK39 were evaluated for their predictive power in the context of Leish+ and Leish- control groups by ROC curve analysis (Supplementary Table S4 and Supplementary Figure S6). The generated ROC curves presented good, SPLA/SECA (AUC = 0.8822) and SPLA/rK39 (AUC=0.8479), and excellent AUC for rK39/SECA (AUC = 0.9545). The ratios on their own have worse sensitivity and specificity than the SPLA and rK39 antigens (Table 2 and Supplementary Table S4).

**Figure 4:**
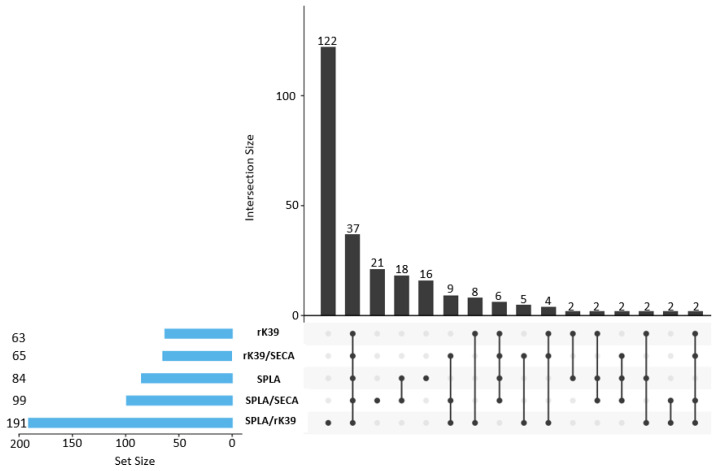
UpSet Plot depicting the positivity intersect to rK39, SPLA, rK39/SECA, SPLA/SECA, and SPLA/rK39 in the PT cohort. The black bars in the graph represent the absolute number of positive events of each intersection, associated with the six parameters evaluated. Under each black bar, the connecting line represents the intersection of the tested parameters. If no intersecting line is present it means that dogs only positive to one event are being quantified in the upper black bar. To the left, the blue bars represent the absolute number of dogs that are positive to each individual characteristic evaluated. In each individual graphic the antigens are ordered by increasing number of seropositive animals.

When the proposed cumulative scoring system was applied to the control cohorts, no overlap in scores existed; the lowest Leish+ score was two while the highest for Leish- was one (Supplementary Figure S7 and Supplementary Table S5). Using this proposed scoring system in the PT cohort resulted in a 26.9% (105/390) seropositivity, considering a score ≥2 (Supplementary Table S5). These 105 dogs include all 63 rK39 seropositive animals and other 42 animals that were seronegative for rK39. It also excluded 16 SPLA seropositive animals. A further nine animals, seronegative for SPLA and rK39, were included in the 105 with a positive score (Figure 3 and Figure 4).

## 4. Discussion

In this study, a cohort of 390 dogs from Portugal CanL endemic regions was clinically evaluated for CS compatible with CanL and tested by DAT and PCR. Then, global seropositivity to six *Leishmania*-specific antigens was assessed using ELISA. The individual seropositivity for the six antigens only varied between 15.6% and 22.3%. Still, only 8.2% of the dogs were seropositive to all antigens, and 38.9% of the cohort was seropositive to at least one antigen. Thus, a 30% variation in seropositivity reporting was possible depending on the used criteria. To clarify the serological profile, a scoring system, using the seroreactivity data for rK39, SPLA, and SECA, was used to reevaluate the cohort. This system identified 26.9% of dogs with serological profiles similar to those found in Leish+ and absent in the Leish- cohort.

The total seroprevalence observed by DAT was 10.5%, while using ELISA, the average seropositivity was 18.1%. The antigens with the higher seropositivity observed were LicTXNPx and SPLA, with 22.3% and 21.5%, respectively. Although the DAT seropositivity was lower when compared to ELISA, the global seropositivity obtained using SPLA 21.5% was in accordance with other reports in the CanL endemic areas of Portugal [17,38,39]. The known limitations associated with DAT methodology can also explain the differences observed, e.g., variability caused by antigen preparation and subjectivity concerning endpoint titre due to naked-eye analysis. The seropositivity associated with the three kinesin-based recombinant antigens was very similar, varying between 15.6 for rKDDR and 17.7% for rK28. Published data with rK28 in dogs support improved sensitivity and specificity in detecting infection not only in clinically diseased dogs but also in clinically healthy infected ones [40,41]. These recombinant antigens are associated with high specificity. Thus, the SPLA seropositivity might be overestimated, as was reported for non-endemic countries [24]. Moreover, the agreement between SPLA and the kinesin antigens in the D and E subgroups was very low, suggesting that SPLA cross-reactivity might be an issue. In fact, considering that SPLA is a complex antigen mixture, the lack of agreement with the kinesin-based antigens is expected. Interestingly, the *k* value associated with the kinesin-based antigens was only substantial (Supplementary Table S3). The lack of agreement was due to seropositivity in dogs without evidence of infection (Supplementary Table S4). In fact, in subgroups D and E there are 18 dogs that are only seropositive for one of these three antigens. This exposes one of the serological test limitations, much like SPLA, even the best recombinant antigens can detect different dogs as seropositive. This was true for all the antigens tested. Of the 390 dogs in this study, 238 dogs were seronegative to all antigens and 152 presented seropositivity to at least one antigen. Of the 152 seropositive dogs, only 21.0% (32/152) had an unequivocal serological profile, being positive for all antigens. On the other hand, 45.4% (69/152) were seropositive to only one antigen. This mixed positivity is likely due to cross-reactivity and/or inadequacy of the cut-offs to detect subclinical infections. All the serological tests performed were set up to detect active disease. In fact, the cohorts used for the ROC curves were dogs with confirmed CanL. The negative cohort was animals from a negative endemic area in Portugal. Thus, the cut-offs determined are defined for the optimal distinction of these two cohorts. All antigens present an excellent capacity to detect CanL with very high Se and Sp. Still, cut-off application to dogs in more complex contexts, their performance is sometimes sub-optimal because they were set up to detect active disease and not for serological surveys. This limitation was shown in a cohort of dogs with clinical suspicion of Canine leishmaniosis, in dogs from non-endemic areas, and in vaccinated animals [23,24,37]. The dog profile that is seropositive to all antigens is clear. Most seropositive dogs to all antigens, 81.2% (26/32), are present in subgroups A and B. For the A cohort, characterized by presenting CS and a positive DAT and/or PCR, this is not unexpected. These are clinically symptomatic dogs similar to those that were used to set up the cut-offs. They present pronounced humoral responses with high titres of anti-*Leishmania* antibodies that are easily detectable by conventional serology. On the contrary, infected animals without clinical signs represent a problem. There is a temporal gap between infection and seroconversion, during this period with often variable serology the cut-offs might not be adequate [10,21]. In the B1 subgroup, only 60% of the dogs were seropositive for LicTXNPx, while 100% were seropositive for SPLA, rK39, rK28, rKDDR, and LAM. LicTXNPx had been reported as a good marker in subclinical infections while being less capable of detecting more advanced infections with clinical signs in both natural and experimentally infected dogs [20,29]. In fact, in experimentally infected dogs, the anti-LicTXNPx antibody profile is characterized by a peak in the early weeks after infection followed by a steady decrease in overall antibody levels [29]. The existence of 12 animals that are positive to all antigens except LicTXNPx supports this notion. This was the most common five antigen combination (only three others existed, two with SPLA absent and one with rK39). The 31 animals that were only seropositive for LicTXNPX suggest that LicTXNPx has a different profile than the other recombinant proteins tested. This anti-LicTXNPx response might be associated with an early humoral response to the parasites. This anti-LicTXNPx response might be associated with an early humoral response to the promastigote forms or the proteins present in the initial inoculum of infection. These might detect very early infections or/ low infection prevalence. In fact, LicTXNPx was found in the in vitro secretome of *L. infantum* both free or vesicle-associated [42]. The fact that LicTXPNx presented the highest overall seropositivity value can also be due to a possible problem of specificity. The ROC analysis suggests that this antigen is less specific. Nonetheless, the pattern of seropositivity was distinct from SPLA. This suggests that the seropositivity to LicTXPNx does not result from the same issue that affects the complex SPLA. In fact, from the 43 dogs detected by LicTXNPx, 39 were seropositive for LicTXNPx and not for SPLA. Ten of the 39 dogs were also seropositive for the antigen mixture LAM, suggesting once again that these LicTXNPx seropositive dogs could be in the early infection stage. SPLA was used above as a comparative for a lack of specificity because this is already observed by us in a previous study [24] using non-endemic animals and also has well-established seroreactivity with other pathogens like *Rickettsia* spp., *Toxoplasma* spp, *Ehrlichia* spp, and other trypanosomatids [18,19,43]. In fact, 17 dogs were only seropositive to SPLA. These dogs, from the D and E subgroups, probably represent animals that have humoral responses to other infections that cross-react with SPLA. Subgroup D was the most interesting group to analyse because it potentially contains undetected subclinical infections. In the D subgroup, LicTXNPx was the antigen associated with a higher percentage of seropositive dogs at 15.4%, followed by SPLA at 10.7%. All the other antigens evaluated presented values of seropositivity between 4.3% and 6.4%. This lower cross-reactivity is expected for the recombinant antigens used in diagnosis, because these are selected for specificity and sensitivity, being less prone to cross-reactivity. Interestingly, for rK39 the seropositivity in the D subgroup is similar to what was reported in a cohort of dogs from non-endemic Europe [24]. This apparent lack of significant cross-reactivity is not supported by the data from the E cohort. This E cohort contains animals with CS but without any evidence of infection. Except for LicTXNPx, all antigens present increased seropositivity compared to the D subgroup. This reactivity associated with the disease is similar to what was reported in CanL dogs suspected [23]. It is worth noting that the percentage of LicTXNPx seropositive dogs did not increase, supporting that cross-reactivity should not be a major contributor to LicTXNPx seroreactivity. Thus, a simple seropositivity evaluation does not provide the most accurate epidemiological information.

An alternative way to address seropositivity was presented in the context of non-endemic and vaccinated animals using not only the absolute seroreactivity but also the relationship between the antigen-specific seroreactivity [24,37]. A model using three antigens, SPLA, rK39, and SECA can be used to look for characteristic serological responses associated with Canine leishmaniosis. This model accounts not only for seropositivity but also for characteristic seroreactivity using the SPLA/SECA, rK39/SECA, and SPLA/rK39 ratios of normalized seroreactivity. Upon ROC analysis using the Leish+ and Leish- cohorts, the defined cut-offs were applied to the PT cohort. These ratios, combined with rK39 and SPLA seropositivity, clarified the serological response of the PT cohort. Using the control cohorts, we defined ≥2 as the score that enabled full separation between the Leish+ and Leish- cohorts. Using this criterion, 26.9% of the PT cohort presented serological profiles that could be associated with CanL. Among these are included all 63 rK39 seropositive dogs and a further 36 rK39 seronegative. This score also excluded 16 SPLA seropositive dogs 16 seronegative for SPLA and rK39. This approach contributed to clarifying complex serological patterns, leading to a better stratification of serological responses by similarity to natural infections.

## 5. Conclusions

Overall, the reported seropositivity levels are similar to those previously reported for Portugal. It also validated the capacity of kinesin-related antigens to detect CanL and active infections, in Portugal. The existence of complex antigen seropositivity patterns that can limit the interpretation of epidemiological information was confirmed. Last but not least, we successfully applied the proposed scoring system using three antigens, SPLA, rK39, and LicTXNPx leading to a better understanding of infection-relevant serological profiles. Approaches similar to the proposed scoring system can be an alternative to conventional serological approaches. The definition of serological profiles can be used to comprehend not only the status of infection but also to integrate complex information like vaccination status or treatment efficacy.

## Figures and Tables

**Table 1:** Classification of PT cohort in groups according to their parasitological, clinical, and serological status.

	A	B		C	D	E
		B1	B2			
CS	≥1	Ø	Ø	Ø	Ø	≥1
DAT	+	+	+	−	Ø	Ø
PCR	+/−	+	−	+	Ø	Ø
(%)	3.3 (13/390)	1.3 (5/390)	5.9 (23/390)	3.3 (13/390)	71.5 (279/390)	14.6 (57/390)

CS: Clinical signs; +: Positive/seropositive’ −: Negative/seronegative Ø: Absent.

**Table 2:** Receiver operating characteristic curve analysis using the Leish+ and Leish- cohorts for the *Leishmania*-specific antigens used in the serological survey.

	SPLA	rLicTXNPx	rK39	rK28	rKDDR	LAM
AUC	1	0.9840	1	1	1	0.9997
(95% CI)	(1.000 to 1.000)	(0.9685 to 0.9996)	(1.000 to 1.000)	(1.000 to 1.000)	(1.000 to 1.000)	(0.9988 to 1.000)
*p* value	<0.0001	<0.0001	<0.0001	<0.0001	<0.0001	<0.0001
Cut-off	0.075	0.040	0.127	0.175	0.098	0.131
Sensitivity (%)	100	94.21	100	100	100	99.17
(95% CI)	(97.00 to 100)	(88.44 to 97.64)	(97.00 to 100)	(97.00 to 100)	(97.00 to 100)	(95.48 to 99.98)
Specificity (%)	100	93.10	100	100	100	100
(95% CI)	(88.06 to 100)	(77.23 to 99.15)	(88.06 to 100)	(88.06 to 100)	(88.06 to 100)	(88.06 to 100)

**Table 3:** General characteristics and seropositivity percentages and absolute numbers, between brackets, in the different cohorts for SPLA, LicTXNPx, rK39, rK28, rKDDR, and LAM. The characteristics CS (clinical signs), DAT (direct agglutination test), and PCR (Positive PCR result for Leishmania genetic material) are labelled as: “+” (positive); “−” (negative); Ø (Absent).

Groups	Characteristic			Antigens					
	CS	DAT	PCR	SPLA	LicTXNPx	rK39	rK28	rKDDR	LAM
PT				21.5 _(84/390)_	22.3 _(87/390)_	16.2 _(63/390)_	17.7 _(69/390)_	15.6 _(61/390)_	15.6 _(61/390)_
A	+	+	+/−	100 _(13/13)_	100 _(13/13)_	100 _(13/13)_	100 _(13/13)_	100 _(13/13)_	100 _(13/13)_
B	Ø	+	+/−	82.1 _(23/28)_	64.3 _(18/28)_	75.0 _(21/28)_	85.7 _(24/28)_	75.0 _(21/28)_	75.0 _(21/28)_
B1	Ø	+	+	100 _(5/5)_	60.0 _(3/5)_	100 _(5/5)_	100 _(5/5)_	100 _(5/5)_	100 _(5/5)_
B2	Ø	+	Ø	78.3 _(18/23)_	65.2 _(15/23)_	69.6 _(16/23)_	82.6 _(19/23)_	69.6 _(16/23)_	69.6 _(16/23)_
C	Ø	Ø	+	20.1 _(3/13)_	38.5 _(5/13)_	20.1 _(3/13)_	15.4 _(2/13)_	30.80 _(4/13)_	20.1 _(3/13)_
D	Ø	Ø	Ø	10.8 _(30/279)_	15.4 _(43/279)_	5.4 _(15/279)_	6.4 _(18/279)_	4.3 _(12/279)_	5.4 _(15/279)_
E	+	Ø	Ø	26.3 _(15/57)_	14.0 _(8/57)_	19.3 _(11/57)_	21.0 _(12/57)_	19.3 _(11/57)_	15.8 _(9/57)_

## Data Availability

Data is contained within the article or Supplementary Material.

## Supplementary Materials

The following supporting information can be downloaded at: https://www.mdpi.com/article/10.3390/microorganisms10102018/s1, Figure S1: Graphical representations of the receiver operating characteristic curves for the *Leishmania*-specific antigens used in the serological survey; Figure S2: Receiver operating characteristic curve for SECA; Figure S3: Representation of seroreactivity in PT group (n = 390) for the different antigens tested; Figure S4: Seroreactivity to the 6 *Leishmania*-specific antigens in all the cohorts from the study; Figure S5: Significance tables for the reactivity to the seven antigens evaluated by OD at 492 between the subgroups using an ordinary one-way ANOVA with Tukey’s multiple comparison test, with single pooled variance; Figure S6: Graphical representations of the receiver operating characteristic curves for the SPLA/SECA, rK39/SECA, and SPLA/rK39 ratios; Figure S7: UpSet Plot depicting the positivity to rK39, SPLA, rK39/SECA, SPLA/SECA, and SPLA/rK39 intersect in the Leish+ cohort; Table S1: Score system from 0 to 6 indicating the cumulative seropositivity to different *Leishmania*-specific antigens (SPLA, rK39, LicTXNPx, rK28, rKDDR, LAM) in the different cohorts from the study; Table S2: Cohen’s kappa coefficient values for the different antigens tested in the PT cohort; Table S3: Cohen’s kappa coefficient values for the different antigens tested in animals without prior evidence of *Leishmania* infection; Table S4: Receiver operating characteristic curve analysis using the Leish+ and Leish- cohorts for the SPLA/SECA, rK39/SECA, and SPLA/rK39 ratios; Table S5: Score system from 0 to 5 indicating the cumulative seropositivity to different *Leishmania*-specific antigens SPLA, rK39, and the SPLA/SECA, rK39/SECA, and SPLA/rK39 ratios in the cohorts from the study.
