# Supplementary material for: Use of Antigen Combinations to Address Complex Leishmania-Seropositivity Patterns in Dogs Living in Canine Leishmaniosis Endemic Regions of Portugal
Source: Microorganisms. 2022 Oct 12;10(10):2018. doi: 10.3390/microorganisms10102018 (PMC9607924; doi:10.3390/microorganisms10102018)
Supplement: Supplementary file 1 [file microorganisms-10-02018-s001.zip › microorganisms-1922318-supplementary.pdf]

microorganisms

MDPI

Article

Use of Antigen Combinations to Address Complex *Leishmania*-Seropositivity Patterns in Dogs Living in Canine Leishmaniosis Endemic Regions of Portugal

Carla Silva Lima [1,2,3], Sofia Esteves [1,2,3], Inês Costa [1,2,3], Hugo Brancal [4], Clara Lima [1,2,3], Célia Amorim [1,2,†], Luís Cardoso [5,6,7,8], Nuno Santarém [1,2,3,*] and Anabela Cordeiro-da-Silva [1,2,3,*]

1. Instituto de Investigação e Inovação em Saúde, Universidade do Porto, 4200-135 Porto, Portugal
2. Instituto de Biologia Molecular e Celular, Universidade do Porto, 4200-135 Porto, Portugal
3. Serviço de Microbiologia, Departamento de Ciências Biológicas, Faculdade de Farmácia da Universidade do Porto, 4050-313 Porto, Portugal
4. Clínica Veterinária da Covilhã, 6200-293 Covilhã, Portugal
5. Faculdade de Ciências da Saúde, Universidade da Beira Interior, 6200-506 Covilhã, Portugal
6. Departamento de Ciências Veterinárias, Escola de Ciências Agrárias e Veterinárias, Universidade de Trás-os-Montes e Alto Douro (UTAD), 5000-801 Vila Real, Portugal
7. Centro de Ciência Animal e Veterinária (CECAV), UTAD, 5001-801 Vila Real, Portugal
8. Laboratório Associado para Ciência Animal e Veterinária (AL4AnimalS), Universidade de Trás-os-Montes e Alto Douro (UTAD), 5000-801 Vila Real, Portugal

* Correspondence: santarem@ibmc.up.pt (N.S.); cordeiro@ibmc.up.pt (A.C.-d.-S.)

† Present address: Departamento de Ciências Químicas, Faculdade de Farmácia da Universidade do Porto, 4200-135 Porto, Portugal.

Supplementary Materials

Supplementary Figures

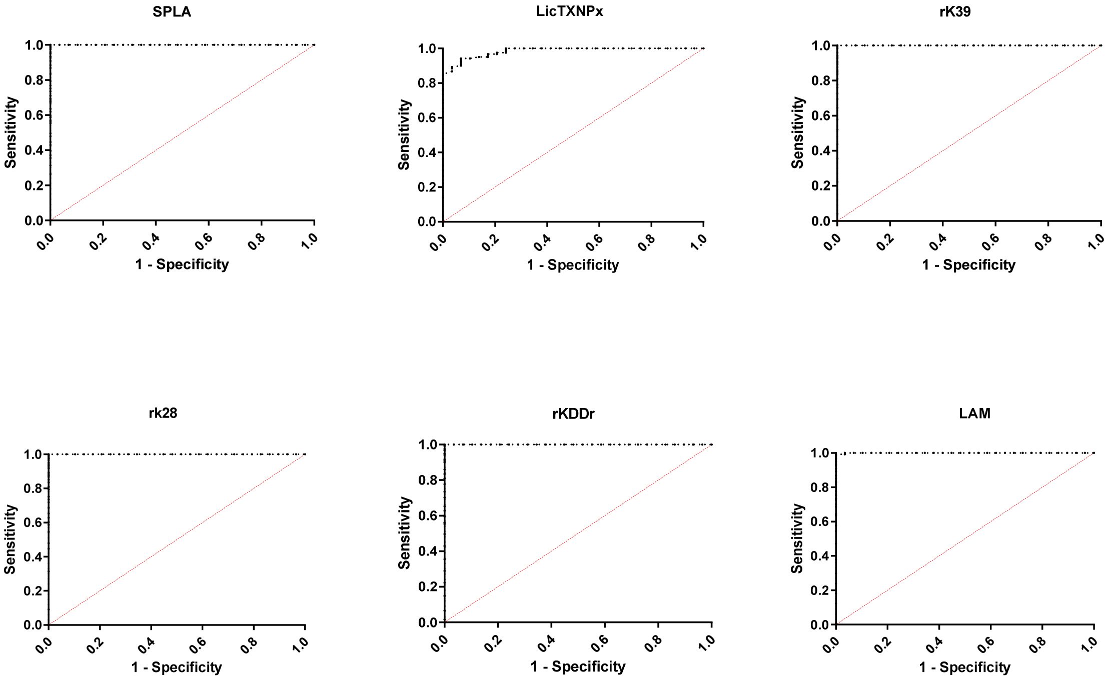

Figure S1. Graphical representations of the receiver operating characteristic curves for the *Leishmania*-specific antigens used in the serological survey.

	SECA
AUC	0.7918
(95% CI)	(0.7103 to 0.8734)
P value	<0.0001
Cut-off	0.126
Sensitivity (%)	70.25
(95% CI)	(61.26 to 78.21)
Specificity (%)	79.31
(95% CI)	(60.28 to 92.01)

A

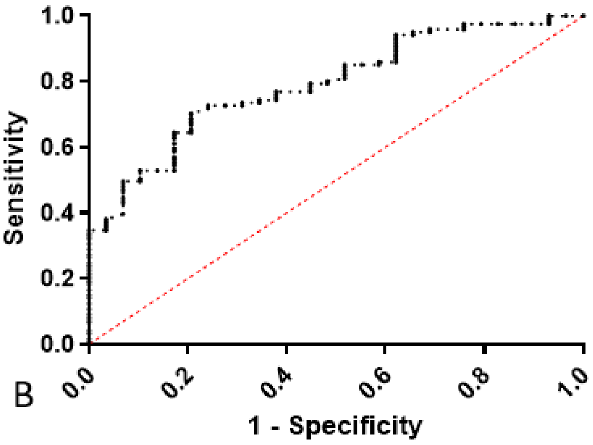

Figure S2. Receiver operating characteristic curve for SECA. (**A**) ROC curve analysis parameters using the Leish+ and Leish− cohorts for the SECA antigens used in the serological survey. (**B**) graphical representation of the ROC curve for SECA.

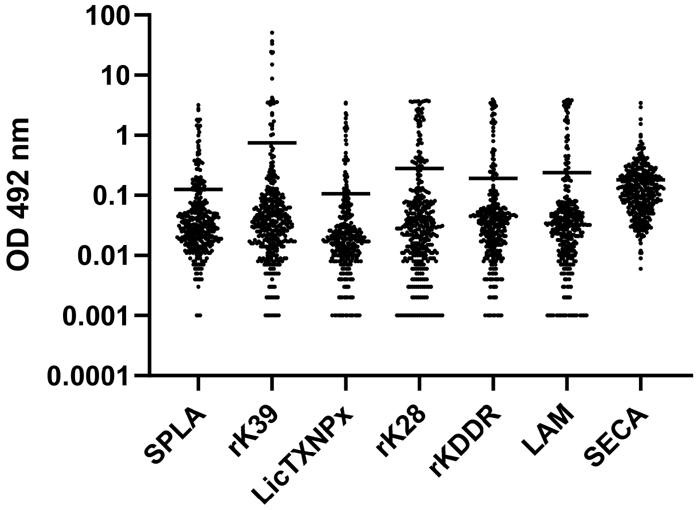

Figure S3. Representation of seroreactivity in PT group (n = 390) for the different antigens tested: SPLA, LicTXNPx, rK39, rK28, rKDDR, LAM and SECA. Results are expressed as the optical density (OD) at 492. Each single dot represents the data set for an individual dog, horizontal bar in each data set represents the median value for the population. The data set is the average of two independent assays done in triplicate.

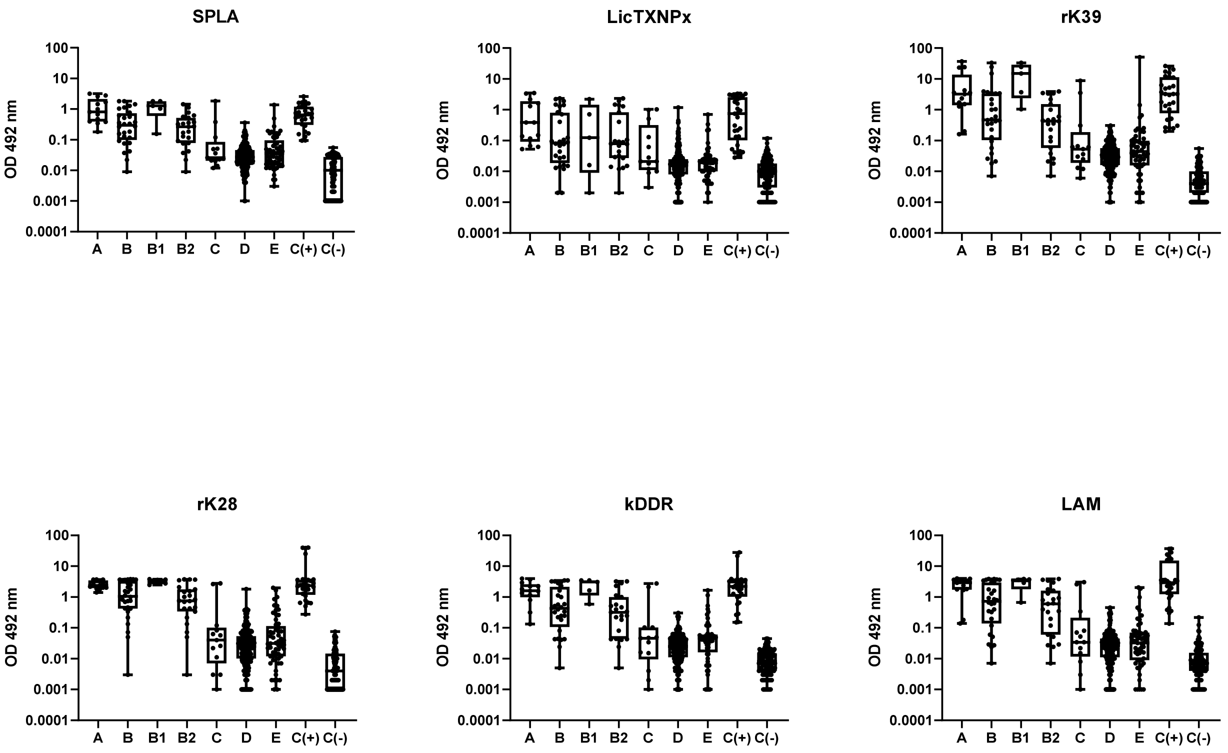

Figure S4. Seroreactivity to the 6 *Leishmania*-specific antigens in all the cohorts from the study. Results are expressed as the optical density (OD) at 492. Each single dot represents the data set for an individual dog. A box and whiskers representation is used to represent the interquartile range and maximum and minimum values for each data set. The horizontal bar in each data set represents the median value for the population. The data set is the average of two independent assays done in triplicate.

SPLA	A	B	B1	B2	C	D	E	Lelsh+	Lelsh-
A		<0.0001	NS	<0.0001	<0.0001	<0.0001	<0.0001	<0.0001	<0.0001
B			<0.0001	NS	NS	<0.0001	<0.0001	0.0026	<0.0001
B1				<0.0001	<0.0001	<0.0001	<0.0001	0.0285	<0.0001
B2					NS	<0.0001	0.0054	<0.0001	<0.0001
C						NS	NS	<0.0001	NS
D							NS	<0.0001	NS
E								<0.0001	NS
Lelsh+									<0.0001
Lelsh-									
Medlan	0.804	0.298	1.286	0.265	0.026	0.027	0.036	0.650	0.010

rK39	A	B	B1	B2	C	D	E	Lelsh+	Lelsh-
A		0.0137	0.0102	<0.0001	0.0001	<0.0001	<0.0001	NS	<0.0001
B			<0.0001	NS	NS	0.0002	NS	NS	0.0005
B1				<0.0001	<0.0001	<0.0001	<0.0001	<0.0001	<0.0001
B2					NS	NS	NS	<0.0001	NS
C						NS	NS	0.001	NS
D							NS	<0.0001	NS
E								<0.0001	NS
Lelsh+									<0.0001
Lelsh-									
Medlan	3.213	0.473	15.12	0.423	0.053	0.031	0.044	3.266	0.004

LicTXNPx	A	B	B1	B2	C	D	E	Lelsh+	Lelsh-
A		0.0037	NS	0.0029	<0.0001	<0.0001	<0.0001	NS	<0.0001
B			NS	NS	NS	<0.0001	0.0002	<0.0001	<0.0001
B1				NS	NS	NS	NS	NS	0.0486
B2					NS	0.0001	0.0024	<0.0001	0.0002
C						NS	NS	<0.0001	NS
D							NS	<0.0001	NS
E								<0.0001	NS
Lelsh+									<0.0001
Lelsh-									
Medlan	0.386	0.084	0.124	0.079	0.021	0.016	0.019	0.744	0.010

rK28	A	B	B1	B2	C	D	E	Lelsh+	Lelsh-
A		NS	NS	NS	NS	0.0414	NS	0.0002	0.0458
B			NS	NS	NS	NS	NS	<0.0001	NS
B1				NS	NS	NS	NS	NS	NS
B2					NS	NS	NS	<0.0001	NS
C						NS	NS	<0.0001	NS
D							NS	<0.0001	NS
E								<0.0001	NS
Lelsh+									<0.0001
Lelsh-									
Medlan	2.361	1.058	3.573	0.758	0.040	0.025	0.031	2.343	0.004

kDDR	A	B	B1	B2	C	D	E	Lelsh+	Lelsh-
A		NS	NS	NS	NS	0.0004	0.0032	0.0055	0.0005
B			NS	NS	NS	0.0092	NS	<0.0001	0.0139
B1				NS	NS	0.006	0.0142	NS	0.0061
B2					NS	NS	NS	<0.0001	NS
C						NS	NS	<0.0001	NS
D							NS	<0.0001	NS
E								<0.0001	NS
Lelsh+									<0.0001
Lelsh-									
Medlan	1.602	0.429	3.073	0.317	0.046	0.025	0.046	2.199	0.008

LAM	A	B	B1	B2	C	D	E	Lelsh+	Lelsh-
A		NS	NS	NS	NS	0.0225	NS	<0.0001	0.0269
B			NS	NS	NS	NS	NS	<0.0001	0.0139
B1				NS	NS	NS	NS	0.0009	0.0061
B2					NS	NS	NS	<0.0001	NS
C						NS	NS	<0.0001	NS
D							NS	<0.0001	NS
E								<0.0001	NS
Lelsh+									<0.0001
Lelsh-									
Medlan	2.912	0.727	3.604	0.602	0.034	0.027	0.032	2.893	0.007

SECA	A	B	B1	B2	C	D	E	Lelsh+	Lelsh-
A		NS	NS	NS	NS	NS	NS	NS	NS
B			NS	NS	NS	NS	NS	NS	NS
B1				NS	NS	NS	NS	NS	NS
B2					NS	NS	NS	NS	NS
C						NS	NS	NS	NS
D							NS	NS	NS
E								NS	0.0284
Lelsh+									0.033
Lelsh-									
Medlan	0.252	0.110	0.251	0.100	0.062	0.118	0.120	0.169	0.087

Figure S5. Significance tables for the reactivity to the seven antigens evaluated by OD at 492 between the subgroups using an ordinary one-way ANOVA with Tukey's multiple comparison test, with single pooled variance.

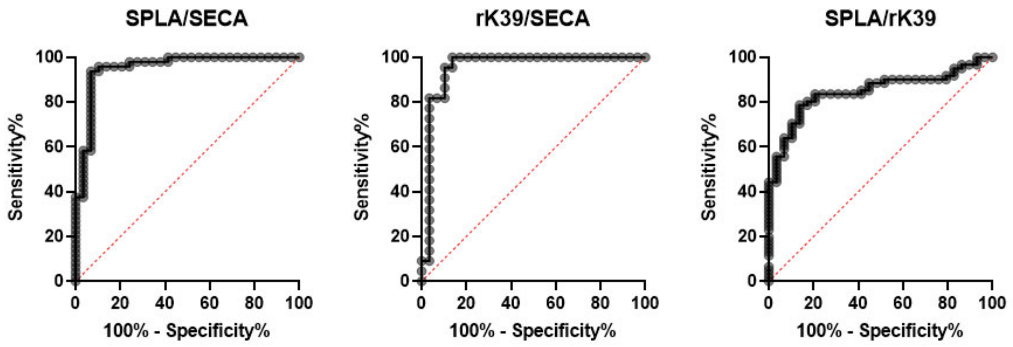

Figure S6. Graphical representations of the receiver operating characteristic curves for the SPLA/SECA, rK39/SECA and SPLA/rK39 ratios.

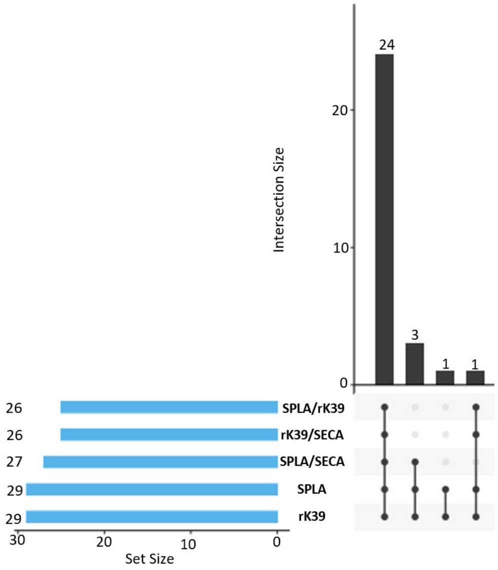

Figure S7. UpSet Plot depicting the positivity intersect to five parameters rK39, SPLA, rK39/SECA, SPLA/SECA, and SPLA/rK39 in the Leish+ cohort. The black bars in the graph represent the absolute number for positive events of each intersection, associated with the six parameters evaluated. Under each black bar, the connecting line represents the in-tersection of the tested parameters. If no intersecting line is present it means that dogs only positive to one event are being quantified in the upper black bar. To the left, the blue bars rep-resent the absolute number of dogs that are positive to each individual characteristic evaluated. In each individual graphic the antigens are ordered by increasing number of seropositive animals.

Supplementary Tables

Table S1. Score system from 0 to 6 indicating the cumulative seropositivity to different *Leishmania*-specific antigens (SPLA, rK39, LicTXNPx, rK28, rKDDR, LAM) in the different cohorts from the study.

		Cumulative seropositivity for the individual antigens						
**Cohort**	**(n)**	**0**	**1**	**2**	**3**	**4**	**5**	**6**
**PT**	**(390)**	238	68	28	5	5	15	32
**A**	**(13)**	0	0	0	0	0	0	13
**B1**	**(5)**	0	0	0	0	0	2	3
**B2**	**(23)**	3	1	1	1	1	6	10
**C**	**(13)**	7	2	1	0	1	0	2
**D**	**(279)**	195	56	20	3	2	3	1
**E**	**(57)**	33	9	6	1	1	4	3
**CanL+**	**(29)**	0	0	0	0	0	2	27
**CanL-**	**(121)**	114	6	1	0	0	0	0

Table S2. Cohen's kappa coefficient values for the different antigens tested in the PT cohort. Values under 0 are considered poor agreement; values between 0 and 0.20 — Slight Agreement; 0.21–0.40 — fair agreement; 0.41–0.60 — moderate agreement; 0.61–0.80 — substantial agreement; 0.81 to 1.00 — almost perfect agreement.

	SPLA	rK39	LicTXNPx	rK28	rKDDR	LAM	SECA
SPLA		0.5914	0.3591	0.6676	0.5538	0.5875	0.0521
rK39			0.3884	0.672	0.7892	0.7316	0.018
LicTXNPx				0.4045	0.4158	0.5475	0.0961
rK28					0.705	0.705	0.0523
rKDDR						0.8058	0.0065
LAM							0.0279
SECA							

Table S3. Cohen's kappa coefficient values for the different antigens tested in animals without prior evidence of *Leishmania* infection. Values under 0 are considered poor agreement; values between 0 and 0.20 — Slight Agreement; 0.21–0.40 — fair agreement; 0.41–0.60 — moderate agreement; 0.61–0.80 — substantial agreement; 0.81 to 1.00 — almost perfect agreement.

	SPLA	rK39	LicTXNPx	rK28	rKDDR	LAM	SECA
SPLA		0.2972	0.3506	0.4176	0.2238	0.2808	0.0157
rK39			0.1173	0.3769	0.5819	0.4383	-0.0127
LicTXNPx				0.1237	0.1644	0.3945	0.0814
rK28					0.4478	0.4368	0.0275
rKDDR						0.5654	-0.0078
LAM							0.0118
SECA							

Table S4. Receiver operating characteristic curve analysis using the Leish+ and Leish− cohorts for the SPLA/SECA, rK39/SECA and SPLA/rK39 ratios.

	**SPLA/SECA**	**rK39/SECA**	**SPLA/rK39**
**AUC**	0.8822	0.9545	0.8479
(95% CI)	(0.7756 to 0.9887)	(0.8906 to 1)	(0.7679 to 0.9280)
**P value**	<0.0001	<0.0001	<0.0001
**Cut-off**	<1.213	<1.646	>1.442
**Se %**	93.75	100	78.69
(95% CI)	(83.16 to 97.85)	(85.13 to 100)	(66.88.00 to 87.10)
**Sp %**	93.1	86.21	86.21
(95% CI)	(78.04.44 to 98.77)	(69.44 to 94.50)	(69.44 to 94.50)

Table S5. Score system from 0 to 5 indicating the cumulative seropositivity to different *Leishmania*-specifc antigens SPLA, rK39, and the SPLA/SECA, rK39/SECA and SPLA/rK39 ratios in the cohorts from the study.

Cohort	(n)	Score					
		0	1	2	3	4	5
PT	(390)	132	153	36	18	14	37
A	(13)	0	0	0	2	0	11
B1	(5)	0	0	0	0	0	5
B2	(23)	2	4	1	1	3	12
C	(13)	2	0	2	1	6	2
D	(279)	106	132	21	13	4	3
E	(57)	22	17	12	1	1	4
CanL+	(29)	0	0	1	3	1	23
CanL-	(121)	121	5	0	0	0	0
